# Elevated galectin-3 in women with gestational diabetes mellitus, a new surrogate for cardiovascular disease in women

**DOI:** 10.1371/journal.pone.0234732

**Published:** 2020-06-17

**Authors:** Yeela Talmor-Barkan, Chava Chezar-Azerrad, Boris Kruchin, Dorit Leshem-Lev, Amos Levi, Eran Hadar, Ran Kornowski, Kinneret Tenenbaum-Gavish, Avital Porter

**Affiliations:** 1 Department of Cardiology, Rabin Medical Center, Petah Tikva, Israel; 2 Sackler Faculty of Medicine, Tel-Aviv University, Tel-Aviv, Israel; 3 FMRC—Felsenstein Medical Center, Petah-Tikva, Israel; 4 Helen Schneider Hospital for Women, Rabin Medical Center, Petah Tikva, Israel; University of Mississippi Medical Center, UNITED STATES

## Abstract

**Background:**

Gestational diabetes mellitus (GDM) is associated with future cardiovascular morbidity and recognized as a women-specific risk factor for cardiovascular disease. The mechanisms for this association are not well established. Therefore, we aimed to evaluate the cardiovascular-related biomarkers, galectin-3 (Gal-3) and protein convertase subtilisin/kexin (PCSK) type 9, in women with GDM.

**Methods:**

Blood samples were drawn in the third trimester from 31 women diagnosed with GDM and from 35 women with normal pregnancies. Blood levels of Gal-3 and PCSK-9 were measured using a quantitative sandwich enzyme immunoassay. In addition, we measured Gal-3 levels in 24 pregnant women in the first trimester who later developed GDM and in 36 healthy controls. Continuous variables were compared using student’s t-test and categorical variables by chi-square/fisher's exact tests.

**Results:**

We found increased levels of Gal-3 in women diagnosed with GDM compared to women without GDM (124.6±32% versus control; pv = 0.001). Furthermore, we demonstrated elevated levels of Gal-3 during the first trimester among women who later developed GDM compared with women who did not develop any gestational morbidity (125.7±32% versus control; pv = 0.004). Third-trimester levels of PCSK-9 did not differ between women with and without GDM (560±45ng/mL versus 553±33ng/mL; pv = 0.4).

**Conclusions:**

The results suggest a possible mechanism that may link GDM to the future increased cardiovascular risk in these patients. Additionally, increased Gal-3 levels during the first trimester may suggest a new early predictor for GDM.

## Background

Heart disease is a leading cause of death among women in the western world. In recent years, awareness of women-specific risk factors has grown, especially pregnancy-related disorders, which were recognized as markers for future cardiovascular disease (CVD) [1].

Gestational diabetes mellitus (GDM) is a common pregnancy complication with increasing incidence due to the global rise of obesity [2]. GDM is associated with short and long term maternal and neonatal complications, specifically, an increased risk of diabetes mellitus (DM) and CVD [3,4]. Currently, GDM diagnosis is made during the late second trimester, possibly exposing both mother and infant to metabolic alterations prior to the diagnosis of GDM [5,6]. Women who are diagnosed with GDM during pregnancy, represent a young and high-risk population for future diabetes and CVD [7]. Previous studies suggested altered lipid metabolism, impaired endothelial function, and vascular inflammation as potential pathways in the pathogenesis of CVD after GDM [1,8,9]. Nevertheless, the underlying mechanisms linking GDM to CVD remain unclear.

Galectin-3 (Gal-3), a versatile protein that belongs to a family of β-galactoside binding proteins, is considered a mediator of cell damage due to its pro-fibrotic and pro-inflammatory properties [10–12]. Gal-3 interacts with cell adhesion molecules and has a high binding affinity for advanced glycation end products mediating free reactive radical production and endothelial dysfunction [12]. Current guidelines for the management of heart failure, recommend obtaining blood levels of Gal-3 as a biomarker for the prediction of both mortality and hospitalization in patients with heart failure [13].

Proprotein convertase subtilisin/kexin (PCSK) type 9 plays an important role in cholesterol homeostasis through its ability to induce degradation of LDL receptors (LDLR) in the lysosome of hepatocytes. Reduced LDLR levels result in decreased metabolism of LDL cholesterol, which leads to hypercholesterolemia [14]. PCSK-9 is mainly expressed in the liver, however, the expression of PCSK-9 was also demonstrated in atherosclerotic plaques [15]. Several studies have suggested a possible pleiotropic effect of PCSK-9 inhibitors in terms of anti-inflammatory properties, besides lowering LDL cholesterol [16,17].

In trying to establish novel biomarkers for early detection of GDM and a possible mechanism for the future development of CVD, we aimed to examine the levels of Gal-3 and PCSK-9 in women with GDM.

## Materials and methods

### Trial participants

Group 1 (G1) included 66 women at 28+0 to 40+0 weeks of gestation (third trimester) that were recruited between November 2016 and January 2020. Thirty-one of them were diagnosed with GDM and 35 had normal pregnancies and served as the control group. Inclusion criteria were an age of 18 years or more and singleton gestation. Exclusion criteria were chronic hypertension, chronic renal disease, coronary heart disease, heart failure, active infectious disease, autoimmune disease, diabetes mellitus type 1 or 2, any pregnancy morbidity other than GDM and major fetal anomalies. The second study group (G2) included 2 cohorts of pregnant women that were recruited at first trimester: (1) The Israeli cohort of the ASPRE trial [18], which originally included 246 pregnant women recruited between April 2014 and August 2017 at 11+0 to 13+6 weeks of gestation (first trimester), of whom 25 developed GDM later during pregnancy. Inclusion criteria for the trial were an age of 18 years or more and a singleton pregnancy. Exclusion criteria were unconscious or severely ill status, learning disabilities or serious mental illness, major fetal abnormalities, regular aspirin treatment within 28 days prior to recruitment, bleeding disorders, peptic ulcerations, hypersensitivity to aspirin, long-term use of nonsteroidal anti-inflammatory medication. The patients were followed until delivery for the development of pregnancy morbidity including GDM. (2) Women with a singleton pregnancy, an age of 18 years or more, at a gestational age of 10–14 weeks, attending the first trimester clinic in the Rabin Medical Center and undergoing the routine nuchal translucency examination since May 2017. The patients were followed until delivery for the development of pregnancy morbidity including GDM. In both G2 group cohorts, we analyzed serum samples taken in the first trimester from women who later developed GDM and from a control group of women who did not develop GDM or other pregnancy morbidity until delivery.

All participants (G1 and G2) were enrolled at the gynecological department at Rabin Medical Center. The recruitment of all participants was approved by the Ethics Committee of Rabin Medical Center and G1 group was also approved by the the Israeli Ministry of Health. In addition, the recruitment of participants from the ASPRE trial was approved by the Investigational Review Board of the ASPRE trial. All subjects provided written informed consent.

### GDM diagnosis

GDM diagnosis was made by a universal two-step approach [3] as follows: All pregnant women underwent an initial screening test with a non-fasting 50g glucose challenge test (GCT) at 24–28 gestational weeks, followed by a fasting 100g glucose tolerance test (GTT) if GCT ≥ 140 mg/dl. GDM was diagnosed when two or more values of the GTT were pathological. Threshold values for GTT were fasting ≥95mg/dl, 1-hour ≥180mg/dl, 2-hour ≥ 155mg/dl and 3-hour ≥ 140mg/dl [19].

### Biomarker measurement

Venous blood was taken from patients in G1 and G2 at the time of recruitment. Gal-3 and PCSK-9 levels were measured in plasma samples of patients in G1. In addition, we measured serum levels of Gal-3 in patients in G2. Venous blood from the enrolled patients was drawn into EDTA-containing tubes or serum separator tubes. All the samples underwent centrifugation at 3000 r/min for 5 min and the collected supernatant was stored at -80°C until biomarker measurement. We used Human Galectin-3 Quantikine enzyme-linked immunosorbent assay (ELISA) Kit, catalog number DGAL30, Lot P212527 (for all kits) from R&D (Minneapolis, MN, USA) according to the manufacturer’s instructions which allows measurments in both serum and plasma. PCSK-9 levels were measured in plasma using ELISA kits from R&D (Minneapolis, MN, USA) according to the manufacturer’s instructions.

N-terminal-pro B-type natriuretic peptide (NT-pro-BNP) and C-reactive protein were measured by high-immunoassay in serum. The concentrations of the plasma triglycerides, total cholesterol, high-density lipoprotein (HDL), low-density lipoprotein (LDL), glucose creatinine and hemoglobin A1C (HbA1C) were measured by automatic biochemistry analyzers.

### Statistical methods

Continuous variables were expressed as mean ± standard deviation and compared using paired Student’s t-test. Categorical variables were compared using chi-square or fisher's exact tests as needed. Receiver operating characteristic (ROC) curve analysis was plotted and area under the curve (AUC) was calculated to evaluate the diagnostic performance of Gal-3 to predict GDM. All analyses were conducted using R: A language and environment for statistical computing, version 3.1.1 (R Foundation for Statistical Computing, Vienna, Austria). p-value <0.05 was considered statistically significant.

## Results

### Patient characteristics

G1 included 66 participants, and G2 included 60 participants. Patient characteristics of the G1 group are shown in Table 1. In this group, the Body Mass Index (BMI) of GDM patients did not significantly differ from the control group. However, patients with GDM had higher rates of previous GDM and higher levels of HbA1C. Table 2 presents the baseline characteristics of participants in G2. The main differences between GDM participants and participants with healthy pregnancies in G2 were BMI and rates of previous GDM which were both higher in women who developed GDM.

**Table 1:** Baseline characteristics of patients in group 1.

	GDM (n: 31)	Normal gestation (n: 35)	*p* value
Age (years)	33.2 ± 5	32.2 ± 4.7	0.4
BMI (kg/m2)	29.3 ± 6.3	28.1 ± 5.6	0.44
Previous GDM [n., (%)]	14 (45%)	3 (8.6%)	0.012
Currently smoking [n., (%)]	1 (3.2%)	1 (2.9%)	1
Gestational age at serum sampling (days)	247.6 ± 22	260 ± 21	0.028
LDL (mg/dL)	97.2 ± 56	106.7 ± 57.3	0.55
HDL (mg/dL)	69 ± 14	66.1 ± 15.7	0.47
Triglycerides (mg/dL)	243.6 ± 67	287.5 ± 110	0.059
HbA1C (%)	5.42 ± 0.5	5.08 ± 0.4	0.004
Creatinine (mg/dL)	0.46 ± 0.1	0.48 ± 1	0.283
Galectin-3 (% of control)	100 ± 25%	124.6 ± 32%	0.001

Data are presented as mean±SD. BMI indicates body mass index; GDM, gestational diabetes; HbA1C, hemoglobin A1C; HDL, high-density lipoprotein; LDL, low-density lipoprotein.

**Table 2:** Baseline characteristics of patients in group 2.

	GDM (n: 24)	Normal gestation (n: 36)	*p* value
Age (years)	34.3 ± 5.4	33.1 ± 4.5	0.36
BMI (kg/m2)	30 ± 6.9	25.4 ± 4	0.005
Nulliparous [n., (%)]	10 (41.7%)	6 (16.7%)	0.065
Previous GDM [n., (%)]	12 (50%)	6 (16.7%)	0.013
Currently smoking [n., (%)]	2 (8.3%)	1 (2.8%)	0.717
Gestational age at delivery (days)	270.4 ± 9	272.8 ± 13	0.41
Neonatal birth weight (gram)	3372 ± 361	3309 ± 492	0.57
Galectin-3 (% of control)	100 ± 30%	125.7 ± 32%	0.004

Data are presented as mean±SD. BMI indicates body mass index; GDM, gestational diabetes.

### Biomarker analysis

**Fig 1:**
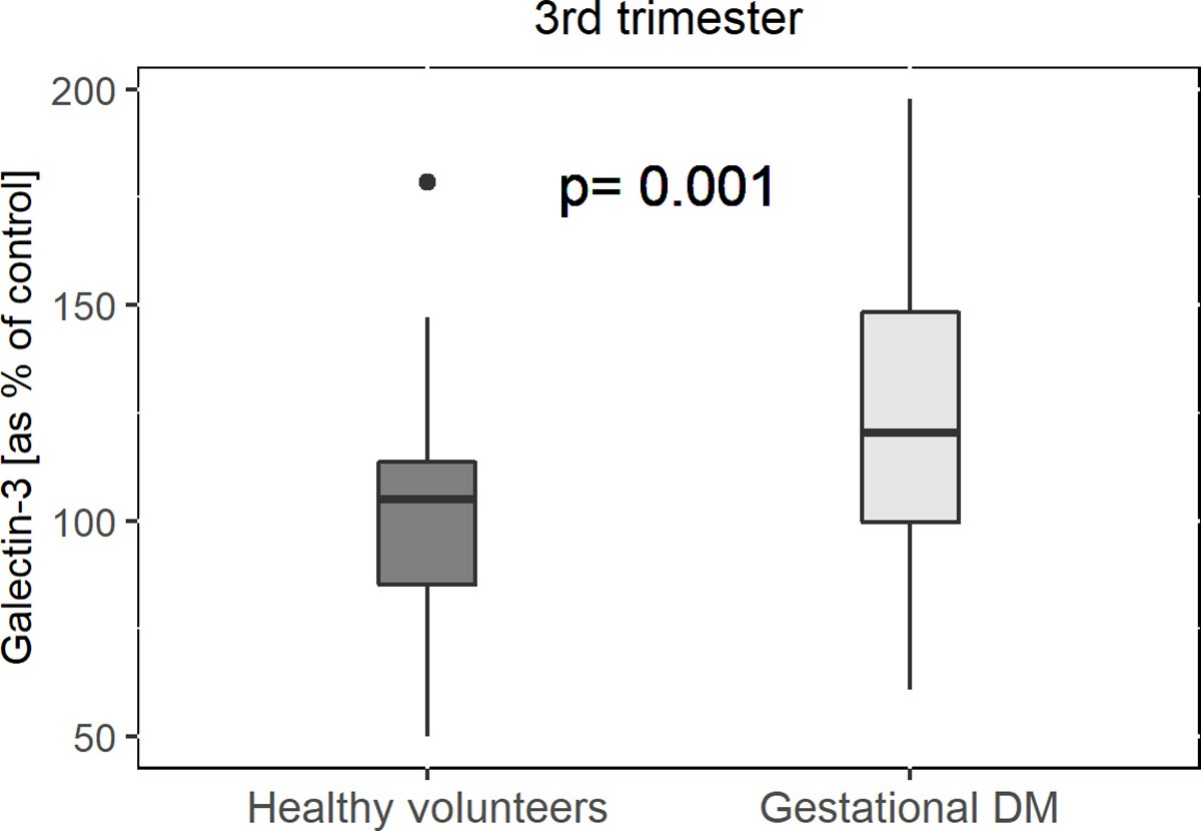
Galectin-3 levels presented as percent of control in women with GDM at 3rd trimester compared to women with normal pregnancy at 3rd trimester. Data are presented in a box plot and includes 31 women with GDM and 35 women with normal pregnancies. The black line within the box marks the median value of galectin-3 presented as percent of control. Galectin-3 was measured using quantitative sandwich enzyme immunoassay.

**Fig 2:**
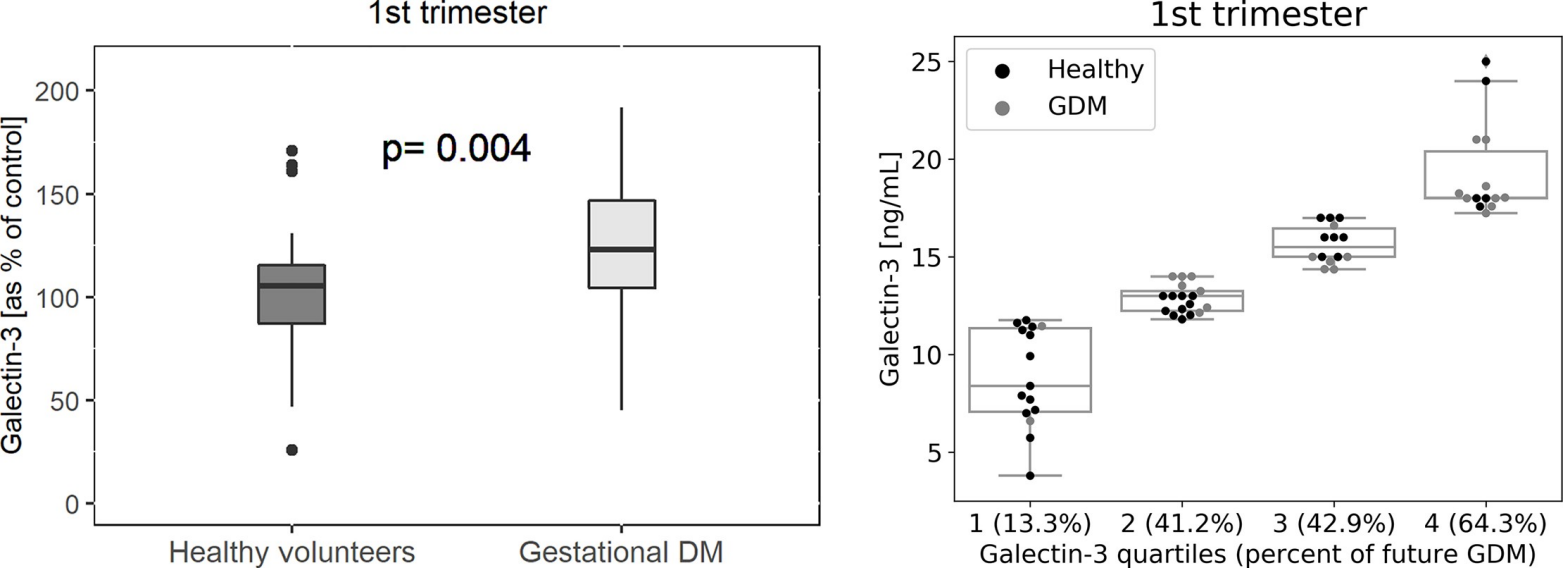
a) Galectin-3 levels presented as percent of control at 1st trimester in women who developed GDM later during pregnancy compared to women with normal pregnancy. Data are presented in a box plot and includes 24 women with GDM and 36 women with normal pregnancies. The black line within the box marks the median value of galectin-3 presented as percent of control. Galectin-3 was measured using quantitative sandwich enzyme immunoassay. b) Percent of women that developed GDM presented in quartiles of galectin-3 levels. Quartiles 1–4 represent different ranges of galectin-3 levels, with rates of women developing GDM during follow-up, presented in parentheses in each quartile.

**Fig 3:**
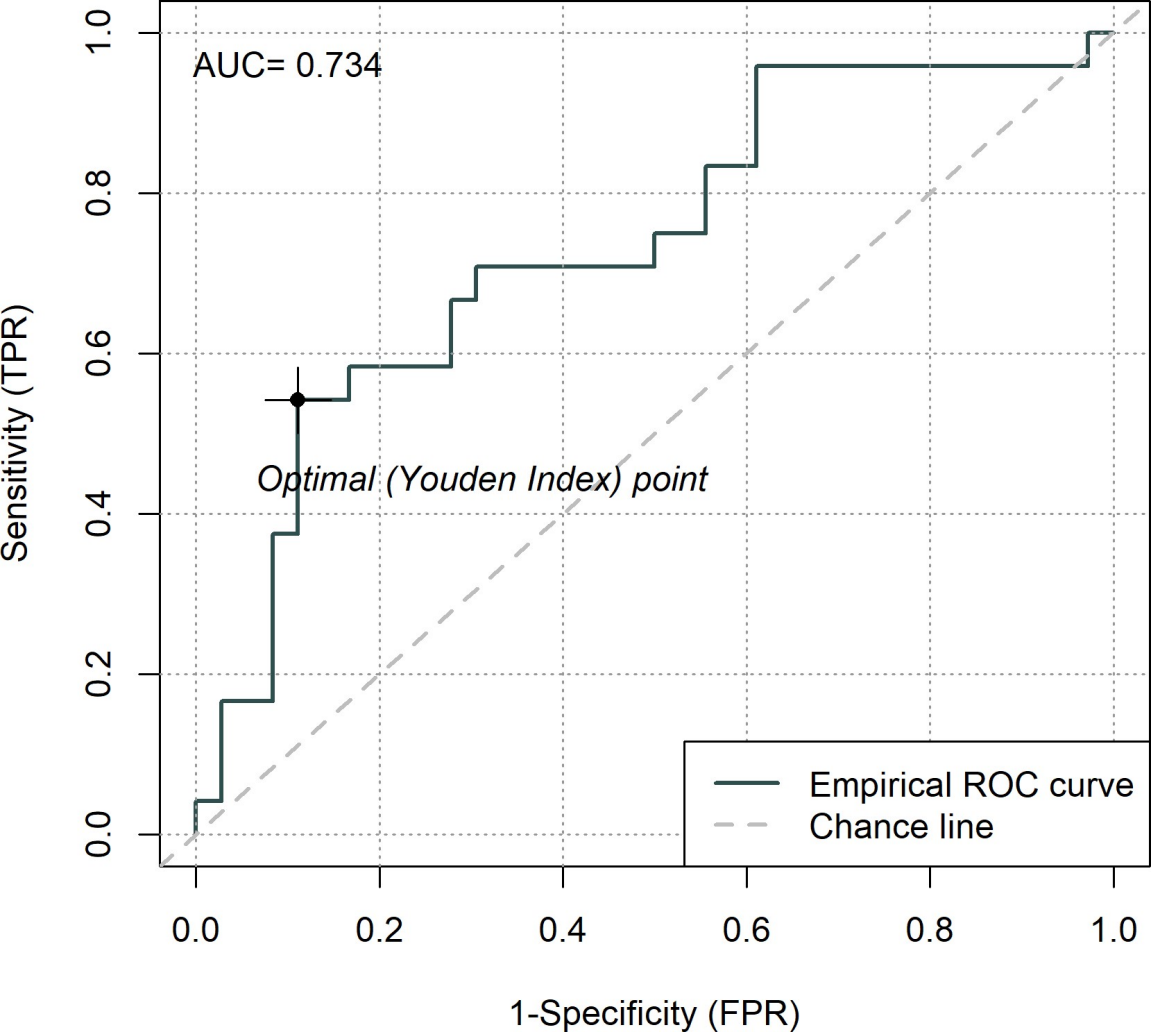
ROC curve analysis and AUC calculation for GDM prediction using galectin-3 levels in first trimester. Receiver operating characteristic (ROC) curve analysis was plotted from galectin-3 levels (percent of control) in first trimester and area under the curve (AUC) was calculated to evaluate the diagnostic performance of galectin-3 to predict GDM.

Blood levels of Gal-3 taken in the third trimester were increased in pregnancies complicated with GDM compared to normal pregnancies (GDM: 100±25% versus healthy pregnancy: 124.6±32%, pv = 0.001; Fig 1). In addition, we demonstrated elevated levels of Gal-3 in pregnant women during the first trimester of women who later developed GDM compared to women who did not develop pregnancy morbidities (GDM: 100±30% versus healthy pregnancy: 125.7±32%, pv = 0.004; Fig 2A). We further divided Gal-3 levels, taken from women in the first trimester, into quartiles and found that in quartile 1 where we had low levels of Gal-3 only 13.3% of women were later diagnosed with GDM. Whereas in the fourth quartile, which had higher levels of Gal-3, 64.3% of all patients later developed GDM (Fig 2B). In addition, ROC curve analysis for serum Gal-3 in first trimester was plotted and the AUC was 0.734 for the prediction of future GDM (Fig 3).

**Fig 4:**
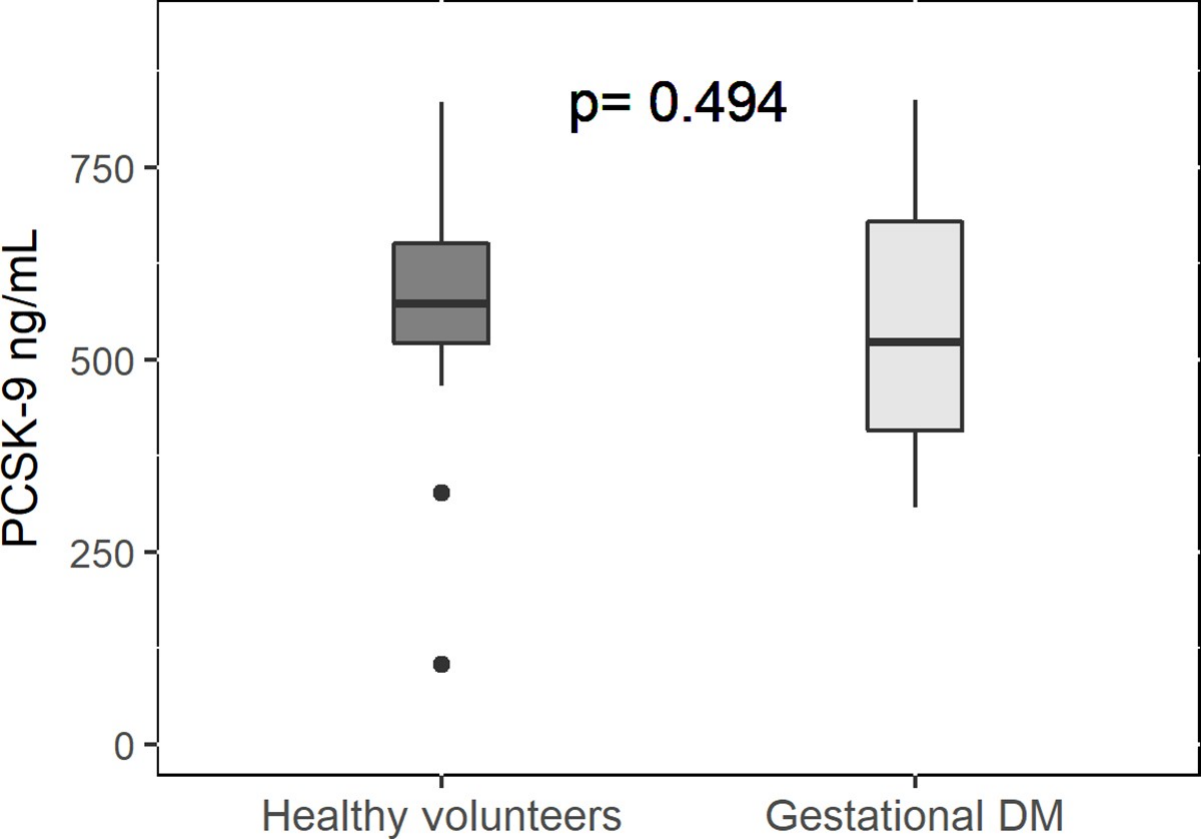
PCSK-9 levels in women with GDM at 3rd trimester compared to women with normal pregnancy at 3rd trimester. Data are presented in a box plot. The black line within the box marks the median value of PCSK-9 level. PCSK-9 was measured using quantitative sandwich enzyme immunoassay.

Plasma levels of PCSK-9 did not differ between healthy volunteers and GDM women in G1 group (GDM: 560±45ng/mL versus healthy pregnancy: 553±33ng/mL, pv = 0.4; Fig 4). No significant differences were observed in NT-pro-BNP, creatinine, HbA1C, LDL, HDL and triglycerides levels, between third-trimester subgroups.

## Discussion

GDM is an important sex-specific cardiovascular risk factor. Women with GDM are at increased risk for both DM and future cardiovascular complications unrelated to the development of DM [7]. The pathophysiology and mechanisms related to those observations are not well established.

In the present study, we hypothesized that blood levels of Gal-3 and PCSK-9 might be related to GDM pathogenesis. We found indeed, a significant association between elevated levels of Gal-3 and GDM diagnosis. Such an association was not found for PCSK-9. As far as we know, our study is the first to demonstrate increased levels of Gal-3 in pregnancies complicated with GDM.

Increased levels of Gal-3 were previously demonstrated in DM and prediabetes patients, with higher Gal-3 levels in diabetes compared to prediabetes patients. In mice, knocking out the Gal-3 gene significantly reduced the development of insulin resistance following a high-fat diet [20]. Previous studies suggested that increased levels of Gal-3 favor the development of insulin resistance and DM [21]. Our results support a possible role of Gal- 3 in the pathogenesis of GDM which could possibly be related to development of DM in the future.

In addition, Gal-3, by various mechanisms, acts as a pro-inflammatory and pro-fibrotic mediator. Genetic disruption of Gal-3 reduces the development of fibrosis in several organs, including the heart and blood vessels. Causality to cardiac injury was demonstrated by the infusion of Gal-3 to pericardial sacs of normal rats, which resulted in increased collagen I/III ratio, which led to cardiac remodeling and dysfunction [10]. We suggest a novel mechanism relating elevated levels of Gal-3 among women with GDM to vascular injury and future CV events. However, this hypothesis should be further evaluated in future studies.

Furthermore, when looking at the women in the first trimester we found that those with higher levels of Gal-3 were more likely to develop GDM later in the pregnancy than women found to have low levels of Gal-3. Early detection of GDM and identifying women at risk for the development of GDM are of major concerns and may dictate early lifestyle interventions and monitoring in order to prevent future morbidity [22,23]. The increased level of Gal-3 during the first trimester, before the development of overt GDM, in women who later developed GDM compared to women with healthy pregnancies may suggest Gal-3 as a potentially novel early biomarker for the development of GDM. Thus, Gal-3 may be used as a laboratory component in risk stratification models for the development of GDM and for the identification of women at high risk for GDM. Based on our findings, the feasibility of using Gal-3 to improve risk stratification models should be evaluated in larger cohorts.

The association between type 2 DM and increased blood levels of PCSK-9 was previously reported in the Dallas Heart Study, which established that plasma levels of PCSK-9 are significantly higher in diabetic patients than in nondiabetic individuals [24]. However, in the present study, we did not find differences in PCSK-9 plasma levels in patients with GDM compared to patients with normal pregnancies.

Our study demonstrated for the first time, an association between elevated blood levels of Gal-3 and newly diagnosed GDM. Moreover, we suggest Gal-3 as a possible biomarker for future development of GDM. Our findings may explain, at least partly, the mechanism of future DM development in GDM women and suggest a possible explanation for the known association between GDM and future CV complications. The novel findings of the present study should be further investigated in larger cohorts.

## Conclusion

We demonstrated that increased levels of Gal-3 in the first trimester may help in the prediction of GDM development later during pregnancy. The increased Gal-3 levels may be involved in the pathogenesis of future GDM complications such as DM and CVD. Further studies are needed to establish the present findings.

## Supporting information

S1 Data (XLSX)
